# Supplementary material for: A single-cell transcriptome atlas of the aging human and macaque retina
Source: Natl Sci Rev. 2020 Aug 25;8(4):nwaa179. doi: 10.1093/nsr/nwaa179 (PMC8288367; doi:10.1093/nsr/nwaa179)
Supplement: nwaa179_Supplemental_Files [file nwaa179_supplemental_files.zip › nwaa179_supplementary_methods_0730.docx]

**Supplementary data**

**The Materials and Methods**

**Tissue sample collection and dissociation**

Human and macaque eye tissue samples were collected in ice-cold artificial cerebrospinal fluid (ACSF) containing 125.0 mM NaCl, 26.0 mM NaHCO_3_, 2.5 mM KCl, 2.0 mM CaCl_2_, 1.0 mM MgCl_2_, 1.25 mM NaH_2_PO_4_; pH 7.4, bubbled with carbogen (95% O_2_ and 5% CO_2_). The sclera, RPE, lens, and vitreous body were then removed. After separation of the whole retina, the retinal fovea (macula) (1.5–2.0 mm) was identified by full of lutein. The peripheral and foveal retina were gently separated into small pieces and centrifuged at 200 g for 2 min. The supernatant was removed, to which was added 500 μl of digestion buffer (2 mg/ml collagenase IV (Gibco), 10 U/μl DNase I (NEB), and 1 mg/ml papain (Sigma) in phosphate-buffered saline (PBS)). The tissue samples were rotated and incubated at 37°C on a thermo-cycler at 300 g for 20–25 min. The sample was pipetted every 5 min [1] to digest the tissue into single cells.

**Library preparation for high-throughput sequencing**

Cells were suspended in 0.04% bovine serum albumin (BSA)/PBS at the proper concentration to generate cDNA libraries with Single Cell 3’ Reagent Kits, according to the manufacturer’s instructions. Thousands of cells were partitioned into nanoliter-scale Gel Bead-In-EMulsions (GEMs) by 10x™ GemCode™ Technology, in which the cDNA produced from the same cell shared a common 10x Barcode. Upon dissolution of the Single Cell 3’ Gel Bead in GEM, primers containing an Illumina R1 sequence (read 1 sequencing primer), 16-bp 10x Barcode, 10-bp randomer, and poly-dT primer sequence were released and mixed with cell lysate and Master Mix. After GEM incubation, barcoded, full-length cDNA from poly-adenylated mRNA was generated. The GEMs were then broken, and silane magnetic beads were used to remove leftover biochemical reagents and primers. Prior to library construction, enzymatic fragmentation and size selection were used to optimize the cDNA amplicon size. P5, P7, an index sample, and R2 (read 2 primer sequence) were added to each selected cDNA during end repair and adaptor ligation. The P5 and P7 primers were used for Illumina bridge amplification of the cDNA (http://10xgenomics.com). Finally, the library was processed on the Illumina HiSeq4000 platform for sequencing with 150-bp pair-end reads.

**Single-cell RNA-seq data preprocessing**

Cell Ranger v2.0.1 (http://10xgenomics.com) was used to process the raw sequencing data with default parameters. Human reads were aligned to the human reference genome (hg19). We used Cell Ranger to create a pre-mRNA reference, using an available transcriptome from the Ensembl genome browser for *M*. *mulatta* (annotation release 95). We excluded poor quality cells after the gene-cell data matrix was generated by Cell Ranger with Seurat (v2.2.0) (https://satijalab.org/seurat/pbmc3k_tutorial.html) in Bioconductor [2, 3]. Only cells that expressed more than 500 genes and fewer than 6 000 genes were considered, and only genes expressed in at least 0.01% of total cells were included for further analysis. Cells with mitochondrial gene percentages over 30% were discarded as well. In total, 22 347 genes across 38 558 human single cells and 18 933 genes across 80 962 macaque single cells remained for subsequent analysis. The data were natural log-transformed and normalized to a total of 1e4 molecules per cell for scaling sequencing depth using Seurat. Batch effects were mitigated using the ScaleData function in Seurat.

**Identification of cell types and subtypes by dimensional reduction**

Seurat (v2.2.0) was used to perform linear dimensional reduction. Highly variable genes with average expression between 0.0125 and 8 and dispersion greater than 1 were selected as inputs for principal component analysis (PCA). We then determined the significant PCs using the JackStrawPlot function. The top 20 PCs were applied for t-Distributed Stochastic Neighbor Embedding (tSNE) to cluster cells with the FindClusters function in Resolution 4.0. Clusters were identified by the expression of known cell-type markers.

**Identification of differentially expressed genes (DEGs) among clusters**

DEG analysis among clusters was performed with the Seurat function FindAllMarkers (thresh.use = 0.25, test.use = “bimod”). The bimod [4] test returns likelihood-ratios for single-cell gene expression, and genes with an average expression difference >0.25 natural log and *P* < 0.05 were selected as marker genes. Enriched Gene Ontology (GO) terms of marker genes were identified using DAVID v6.7 [5, 6] (https://david.ncifcrf.gov/home.jsp).

**LIGER analysis for human and macaque datasets**

To evaluate the conservation and variation between the human and macaque retina transcriptomes, we used LIGER [7] (https://macoskolab.github.io/liger/) to integrate human and macaque retina datasets with the function createLiger. The function selectGenes (var.thresh = 0.1) was used to perform variable gene selection on human and macaque datasets separately and then in combination. We next identified cells loaded on corresponding cell factors and quantile-normalized their factor loadings across datasets. Cell dimensionality reduction was performed with the function runTSNE. The function plotGeneLoadings was used to visualize the most highly loaded genes (both shared and dataset-specific) for each factor. To compare different cluster assignments, we employed the function makeriverplot to visualize the previous cell-type assignments of humans and macaques with liger joint clusters.

**Gene expression correlation analysis between human and macaque cell types**

To explore the similarities between human and macaque cell types, we calculated the Pearson correlation coefficients across humans and macaques with shared variable genes in both datasets. The resulting correlation matrices were visualized with circus plot using the R Package circlize.

**WGCNA in regional DEGs of human MGs**

To identify the human MG regionally related gene modules, we identified the DEGs between foveal and peripheral MG, and then obtained various gene modules under WGCNA [8, 9] (https://cran.r-project.org/src/contrib/Archive/WGCNA). Module assignment was followed by quantifying the relationship between modules and region traits, where the correlations among them were computed and shown as a heatmap. The blue module had the closest association with the fovea. The blue module network was plotted using Cytoscape.

**Co-expression analysis for aging-related genes**

We divided the samples into adult (Y35, Y52, Y63) and aged groups (Y86, Y87). We then performed FindAllMarkers using the Seurat package to identify DEGs. Only genes with an average expression difference greater than 0.5 were selected as aging-related genes. The up-regulated (enriched in aged group) and down-regulated (enriched in adult group) age-related genes were calculated by mean expression among different aging stages, respectively.

**Intercellular network analysis**

Cell-cell interactions were predicted using a method similar to that described previously [10, 11]. We created a cell communication interactome and collected known protein-protein interactions between receptor and ligand and all related genes were collected [11]. Gene lists were manually filtered with the DEGs of the adult to aged groups in our cell types. To investigate aging-related perturbations in the putative cell-cell interaction networks, DEG metrics from the MAST analysis outlined above were used to build subnetworks for each set of interactions between cell types. In these networks, nodes represent ligands or receptors expressed in the denoted cell type, and edges represent protein-protein interactions between them. Nodes were color-coded to represent the magnitude of DEGs. These values were scaled per cell type and summed to determine edge weight.

**Assignment of pseudo-aging score for human retina cells**

Aging-related genes were downloaded from Human Ageing Genomic Resources (https://genomics.senescence.info/). We performed PCA with aging-related genes using the function RunPCA and determined statistically significant PCs using the function JackStraw. We then computed the correlation between the age vector and significant PCs and then selected the PC resulting in the highest correlation coefficient. Pseudo-aging scores of cells were determined by the average expression of the chosen PC genes.

**Analysis of cell-type specific expression of diseases**

The human retina disease genes were obtained from the Retinal Information Network (<http://www.sph.uth.tmc.edu/RetNet/>). For each of the foveal and peripheral cell types that belonged to humans and macaques, we first calculated the expression of all genes across all cell types and computed the fraction of cells in each cell type. We then obtained a matrix of gene expression scores for all genes across all cell types, and selected retinal disease related genes to visualize expression patterns. We computed the mean relative expression strengths among each cell type for the different retinal diseases (e.g., night blindness, macula dystrophy, rod cone dystrophy, dominant RP, recessive RP, AMD, recessive achromatopsia). The calculation method was described by Peng et al.[12]. Finally, we calculated *P* values across different cell types for each disease gene by using bootstrap hypothesis test.

**ATAC library preparation for high-throughput sequencing**

ATAC-seq was performed as described previously [13, 14]. In total, 50,000–60,000 cells were twice washed with 50 μl of cold PBS and [immediately](javascript:;) resuspended in 50 μl of ATAC-lysis buffer (10 mM Tris-HCl pH 7.4, 10 mM NaCl, 3 mM MgCl_2_, 0.1% (v/v) Nonidet P40 Substitute) and centrifuged for 10 min at 500 g at 4 ℃. The resulting supernatant was then removed and added to 50 μl of transposition reaction mix (10 μl 5 × TTBL buffer, 4 μl TTE Mix, and 36 μl nuclease-free H_2_O) of a TruePrep DNA Library Prep Kit V2 (Vazyme TD501-02). Samples were then incubated at 37 ℃ for 30 min. After the reaction finished, [deoxynucleotide](javascript:;) was isolated using a QIAquick PCR Purification Kit (QIAGEN 28106). The ATAC-seq libraries were then prepared using the Trueprep DNA Library Prep kit V2 (Vazyme TD501-02). Finally, 2 × 150 paired-end sequencing was performed on an Illumina HiSeq X-10.

**ATAC-seq data analysis**

We cleaned the reads using fastp (v0.19.6) and then mapped the reads to the hg19 reference genome with the parameters: -t -q -N 1 -L 25 -X 2000 using Bowtie2 (v2.3.4.3). All unmapped reads, non-uniquely mapped reads, and polymerase chain reaction (PCR) duplicates were removed. Fragments mapped to blacklisted genomic regions were removed. The uniquely mapped reads were shifted +4/−5 bp according to the strand of the read. To visualize the ATAC-seq signal, we extended each read by 50 bp and counted the coverage for each base. All ATAC-seq peaks were called by MACS2 v2.1.1.

**ATAC-seq data quality control**

Quality of the ATAC-seq data was evaluated for several parameters, including number of raw reads, alignment rate, percentage of reads mapped to chromosome M, percentage of reads mapped to repeat regions (black list), percentage of reads passed MAPQ score filter, percentage of total signal within known artefact regions, and correlation between replications.

**Connecting transcription factors to target genes**

To find the potential transcription factors binding to *MYO9A*, the *THRB* regulatory sequence, FIMO from MEME Suite (v5.0.5) was used for motif enrichment analysis with default parameters.

**Immunohistochemistry**

Human retinal tissue samples were fixed overnight in 4% paraformaldehyde. The fixed retinae were dehydrated in 20% and 30% sucrose in PBS at 4 °C and embedded in optimal cutting temperature medium (Thermo Scientific). Thin 20–25 μm cryosections were collected on superfrost slides (VWR) using a Leica CM3050S cryostat. For immunohistochemistry, antibodies against the following proteins were used at the indicated dilutions: Mouse anti-RLBP1 (1:500, Abcam), Mouse anti-Rod-OPSIN (1:1 000, Sigma), Rabbit anti-S-OPSIN (1:500, Millipore), Rabbit anti-L/M-OPSIN (1:500, Millipore), Mouse anti-Calbindin (1:500, Abcam), Rabbit anti RRKCA (1:500, Abcam), Sheep anti VSX2 (1:400, Exalpha Biologicals), Sheep anti ONECUT2 (1:40 , R&D Systems), Goat anti OTX2 (1:200 R and D Systems), Rabbit anti-RCVRN (1:500, Millipore), Rabbit anti-NPVF (1:500, Sigma), Rabbit anti-TRH (1:500, Sigma), Rabbit anti BTG1 (1:100, SAB biotech), Mouse anti NR2E3 (1:100, R&D Systems) and Goat anti IBA1 (1:500 Abcam). Primary antibodies were diluted in blocking buffer containing 10% donkey serum, 0.2% Triton X-100, and 0.2% gelatin in PBS at pH 7.4. Alexa Fluor 488, Alexa Fluor 594, or Alexa Fluor 647 fluorophore-conjugated secondary antibodies (1:500) (Life Technologies) were used as appropriate. Cell nuclei were stained with DAPI (1:10 000). Images were collected using an Olympus FV1000 and Olympus FV3000 confocal microscope (Japan).

**RNAscope**

RNAscope® [detection](javascript:;) was conducted [in](javascript:;) [strict](javascript:;) [accordance](javascript:;) [with](javascript:;) the ACD RNAscope® protocols [15]. [Fresh](javascript:;) retinal sections were dehydrated in sequential incubations with ethanol, and then repaired in boiling repair solution for 5 min, followed by 30 min protease III treatment and washing in ddH_2_O. Appropriate combinations of hybridization probes (CYP26A1 Cat#487741; MYO9A Cat#518511; RLBP1 Cat#414221-C2; DIO2 Cat#562211-C3; LHX1 Cat#493021; ISL1 Cat#478591-C2)were incubated for 2 h at 40 °C, followed by fluorescence labeling, DAPI counterstaining, and mounting with Prolong Gold mounting medium.

**References**

1. Zhong, S., et al., *A single-cell RNA-seq survey of the developmental landscape of the human prefrontal cortex.* Nature, 2018. **555**(7697): p. 524-528.

2. Macosko, E.Z., et al., *Highly Parallel Genome-wide Expression Profiling of Individual Cells Using Nanoliter Droplets.* Cell, 2015. **161**(5): p. 1202-1214.

3. Satija, R., et al., *Spatial reconstruction of single-cell gene expression data.* Nat Biotechnol, 2015. **33**(5): p. 495-502.

4. McDavid, A., et al., *Data exploration, quality control and testing in single-cell qPCR-based gene expression experiments.* Bioinformatics, 2013. **29**(4): p. 461-7.

5. Huang, D.W., B.T. Sherman, and R.A. Lempicki, *Systematic and integrative analysis of large gene lists using DAVID bioinformatics resources.* Nature Protocols, 2009. **4**(1): p. 44-57.

6. Huang da, W., B.T. Sherman, and R.A. Lempicki, *Bioinformatics enrichment tools: paths toward the comprehensive functional analysis of large gene lists.* Nucleic Acids Res, 2009. **37**(1): p. 1-13.

7. Welch, J.D., et al., *Single-Cell Multi-omic Integration Compares and Contrasts Features of Brain Cell Identity.* Cell, 2019. **177**(7): p. 1873-1887 e17.

8. Langfelder, P. and S. Horvath, *WGCNA: an R package for weighted correlation network analysis.* Bmc Bioinformatics, 2008. **9**.

9. Langfelder, P. and S. Horvath, *Fast R Functions for Robust Correlations and Hierarchical Clustering.* J Stat Softw, 2012. **46**(11).

10. Kirouac, D.C., et al., *Dynamic interaction networks in a hierarchically organized tissue.* Mol Syst Biol, 2010. **6**: p. 417.

11. Ximerakis, M., et al., *Single-cell transcriptomic profiling of the aging mouse brain.* Nat Neurosci, 2019. **22**(10): p. 1696-1708.

12. Peng, Y.R., et al., *Molecular Classification and Comparative Taxonomics of Foveal and Peripheral Cells in Primate Retina.* Cell, 2019. **176**(5): p. 1222-1237 e22.

13. Buenrostro, J.D., et al., *ATAC-seq: A Method for Assaying Chromatin Accessibility Genome-Wide.* Curr Protoc Mol Biol, 2015. **109**: p. 21 29 1-21 29 9.

14. Buenrostro, J.D., et al., *Transposition of native chromatin for fast and sensitive epigenomic profiling of open chromatin, DNA-binding proteins and nucleosome position.* Nat Methods, 2013. **10**(12): p. 1213-8.

15. Wang, F., et al., *RNAscope: a novel in situ RNA analysis platform for formalin-fixed, paraffin-embedded tissues.* J Mol Diagn, 2012. **14**(1): p. 22-9.

**Figure legends**

**Supplementary Figure 1. Single-cell RNA-seq information and molecular diversity of primate retina.**

(a) Schematic of major cell types and three-layer construction in retina.

(b) Quality control for human and macaque samples, with each dot representing a single cell (F: Fovea; P: Periphery).

(c) t-SNE visualization of human retina samples colored by cluster (left), age, and region (right). Each dot represents an individual cell.

(d) Expression patterns of known markers for different cell types in human adult retina displayed in t-SNE plots (gray, no expression; red, relative expression).

(e) Violin plots showing expression of different cell-type marker genes, distinguishing 56 subclasses in human adult retina.

(f) t-SNE visualization of macaque retina samples colored by cluster (left), age, and region (right). Each dot represents an individual cell.

(g) Expression patterns of known markers for different cell types in macaque adult retina displayed in t-SNE plots.

(h) t-SNE visualization of 119 520 single cells analyzed by LIGER, color-coded by LIGER joint clusters (left) and species and regions (right) (H-F: human-fovea; H-P: human-periphery; M-F: macaque-fovea; M-P: macaque-periphery).

(i) Cell factor loading values (left) and gene-loading plots (right) of LIGER joint clusters and shared or species-specific genes for factor 6 and factor 18.

**Supplementary Figure 2. Distinct subtypes of human and macaque bipolar cells.**

(a) Violin plots showing expression of marker gene *OTX2*, distinguishing 17 rod subclasses in human adult retina.

(b) Left: confocal imaging of *MYO9A* in adult macaque retina. Solid arrowheads indicate *MYO9A^+^* cells; empty arrowheads indicate *MYO9A^-^* cells. Blue, DAPI (nucleus marker). Scale bar, 10 μm (top), 5 μm (bottom). Experiments were repeated three times independently with similar results. Right: Bar chart showing the quantification of Fig. 2d and supplementary Fig. 2b. Data are means ± s.e.m. Each sample was counted from three different slices.

(c) Left: confocal imaging of OTX2 in adult macaque retina. Solid arrowheads indicate OTX2^+^ cells; empty arrowheads indicate OTX2^-^ cells. Blue, DAPI (nucleus marker). Scale bar, 10 μm (left), 5 μm (right). Experiments were repeated three times independently with similar results. Right: Bar chart showing the quantification of Fig. 2f (Y-52 periphery) and supplementary Fig. 2c. Data are means ± s.e.m. Each sample was counted from three different slices.

(d) Dot plot for broad markers and type-enriched markers of different bipolar cell clusters in human adult retina. Color of each dot shows average scale expression, and its size represents percentage of cells in cluster.

(e) Dot plot for type-enriched markers of BB/GB subclasses in human and macaque adult retina. Color of each dot shows average scale expression, and its size represents percentage of cells in cluster.

(f) Dot plot for broad markers and type-enriched markers of different bipolar cell clusters in macaque adult retina. Color of each dot shows average scale expression, and its size represents percentage of cells in cluster.

(g) Dot plot for broad markers and type-enriched markers of different bipolar cell subclasses in human (red) and macaque (green) adult retina. Size of each dot represents percentage of cells in each cluster. Gray to red/green indicates gradient from low to high gene expression.

**Supplementary Figure 3. Molecular properties of primate MGs and cones.**

(a) t-SNE plot of human Müller glia distinguished by regions (dots, single cell; color, regions).

(b) Violin plots showing expression of DEGs that distinguish Müller glia subclasses in human adult retina.

(c) Gene dendrogram obtained by average linkage hierarchical clustering. Color underneath row corresponds to module assignment; each color represents different assigned module.

(d) Module-trait associations. Each row corresponds to a module eigengene, column to a trait. Each cell contains corresponding correlations and *P* values. Color key from blue to red indicates low to high correlation.

(e) *In situ* RNA hybridization of *CYP26A1* and *RLBP1* in Y58 human retina. Blue, DAPI (nucleus marker). Scale bar, 50 μm (left), 10 μm (right).

(f) t-SNE plot of macaque Müller glia distinguished by regions (dots, single cell; color, regions).

(g) Volcano plot for DEGs of macaque foveal and peripheral Müller glia. Red dots show average log2 fold changes >0.5.

(h) Normalized ATAC-seq profiles of *RFC1*, *POLE4*, and *APLP2* in Y27 foveal retina showing activation of these genes. DNA binding motif of *THRB* (top), identified in ATAC-seq peaks close to *THRB* transcription start site (TTS).

(i) Immunostaining of L/M -OPSIN/CALBINDIN and S-OPSIN/CABINDIN in Y54, Scale bar, 300 μm. Experiments were repeated three times independently with similar results.

**Supplementary Figure 4. Single-cell profiles of retinal horizontal cells in primates.**

(a) Visualization of expression of known marker genes for horizontal cells by t-SNE in human adult retina. Cells are colored according to gene expression levels.

(b) Visualization of expression of known marker genes for horizontal cells by t-SNE in macaque retina. Cells are colored according to gene expression levels.

(c) Visualization of expression of *PCDH9* for human (left) and macaque (right) horizontal cells by t-SNE. Cells are colored according to gene expression levels.

**Supplementary Figure 5. Cell-type-specific aging patterns in human retina and aging-related changes in cell-cell communications.**

(a-h) Enriched GO terms of cell type specific aging up/down-regulated genes. a(Rod), b(Cone), c(AC), d(BC), e(GC), f(HC), g(MG), h(Others).

(i) Immunostaining of PRKCA and VSX2 in human Y52 and Y87 peripheral retina. Solid arrowheads indicate double positive cells; empty arrowheads indicate PRKCA negative cells. Scale bar, 25 μm (left), 10 μm (right). Experiments were repeated three times independently with similar results.

(j) Bar chart showing quantification of Supplementary Fig. 5i. Data are means ± s.e.m, *P* values calculated by two-sided *t*-test. n.s., no significance. Each sample was counted from three different slices.

(k) Proportion of rod subclasses (*MYO9A*^+^ and *MYO9A*^-^) in total human retinal cells at each stage.

(l) Aging-related ligands produced and secreted by rods with receptors expressed in MGs (left) and aging-related ligands produced and secreted by rod with receptors expressed in BCs (right).

(m) Aging-related ligands produced and secreted by HCs with receptors expressed in rods (left) and aging-related ligands produced and secreted by microglia with receptors expressed in rods (right). In (l) and (m) panels, nodes represent ligands or receptors expressed in denoted cell type, and edges represent protein-protein interactions between them. Node color represents magnitude of DEGs. Edge color represents sum of scaled differential expression magnitudes from each contributing node, whereas width and transparency are determined by magnitude of scaled differential expression. These figures have been filtered such that top 100 edges representing most differentially expressed node pairs are shown.

(n) Bar chart showing quantification of Fig. 5o. Data are means ± s.e.m, P values calculated by two-sided *t*-test. n.s., no significance, ****P*<0.001, *****P*<0.0001, each sample was counted from three different slices.

**Supplementary Figure 6. Trajectories of human retinal aging.**

(a) Trajectories of expression of classical human aging genes across human adult retina aging pseudotime. The shadow represents the confidence interval (95%) around the fitted curve.

(b-c) Trajectories of expression of up-regulated (b) and down-regulated (c) genes across human adult retina aging pseudotime. The shadow represents the confidence interval (95%) around the fitted curve.

(d-e) Average expression of genes enriched in foveal (d) and peripheral MGs (e).

(f) Cell proportions of macaque H1 and H2 subclasses at different aging stages.

**Supplementary Figure 7. Expression patterns of genes in humans and macaques related to human retinal diseases.**

(a-b) Expression patterns of human eye disease-associated genes in human (a) and macaque (b) retinal cell types. AMD: age-related macular degeneration. CCCRD: choroideremia, cone and cone-rod dystrophy. CCRD: cone and cone-rod dystrophy. DCD: dominant cone dystrophy. DCRD: dominant cone-rod dystrophy. DCSNB: dominant congenital stationary night blindness. DFEV: dominant familial exudative vitreoretinopathy. SR: somatic retinoblastoma. DMDS: dominant macular dystrophy, Stargardt-like. DMDV: dominant macular dystrophy, vitelliform. DMRD: dominant Martinique retinal dystrophy and retinitis pigmentosa. DNIV: dominant neovascular inflammatory vitreoretinopathy. DOMD: dominant occult macular dystrophy. DRMD: dominant radial macular drusen. DRCS: dominant renal-coloboma syndrome. DRP: dominant retinitis pigmentosa. DSS: dominant Stickler syndrome. DWDEM: dominant Wagner disease and erosive vitreoretinopathy. OA: optic atrophy. OED: Oregon eye disease. RAB: recessive abetalipoproteinemia. RAC: recessive achromatopsia. RBBS: recessive Bardet Biedl syndrome. RBFR: recessive benign fleck retina. RBCCD: recessive Bietti crystalline corneoretinal dystrophy. RBSCD: recessive Boucher-Neuhauser syndrome with chorioretinal dystrophy. RCM: recessive chorioretinopathy and microcephaly. RCCRD: recessive cone and cone-rod dystrophy. RCD: recessive cone dystrophy. RCRD: recessive cone-rod dystrophy. RCSNB: recessive congenital stationary night blindness. RDCA: recessive delayed cone adaptation. RGA: recessive gyrate atrophy. RJS: recessive Jobert syndrome. RLCA: recessive Leber congenital amaurosis. RMC: recessive microcephaly with chorioretinopathy. RMGFR: recessive microcephaly, growth failure and retinopathy. RNRD: recessive nephronophthisis with retinal degeneration. RNSD: recessive non-syndromic deafness. ROD: recessive Oguchi disease. ROA: recessive optic atrophy. RPDE: recessive pseudoxanthoma elasticum. RFD: recessive refsum disease. RRD: recessive retinal disease. RRDT: recessive retinal dystrophy. RRP: recessive retinitis pigmentosa. RSLS: recessive Senior-Loken syndrome. RSPNOA: recessive spastic paraplegia, neuropathy, and optic atrophy. RSD: recessive Stargardt disease. RSNSRD: recessive syndromic and non-syndromic retinal dystrophy. RSDRP: recessive syndromic disease with retinitis pigmentosa. RUS: recessive Usher syndrome. RWS: recessive Wolfram syndrome. RPDNA: retinitis pigmentosa with developmental and neurological abnormalities. RTH: retinoschisis. *P* values were calculated by bootstrap hypothesis test, **P* <0.05, ** *P* <0.01, *** *P* <0.001, *****P*<0.0001.

**Supplementary Table1. Retina sample information.** Spreadsheet includes human and macaque adult retinal sampling information.

**Supplementary Table2. Top 100 DEGs of different cell types of human retina.** Spreadsheet includes 100 marker genes of all 10 major cell types of human retina.

**Supplementary Table3. Top 100 DEGs of different cell types of macaque retina.** Spreadsheet includes 100 marker genes of all nine major cell types of macaque retina.

**Supplementary Table 4. Up- and down-regulated genes of human retinal aging.** Spread sheets include 87 and 121 up- and down-regulated genes, respectively, when human retina age.

**Supplementary File 1**. **Codes for bioinformatic analysis.**
