# Supplementary figures and images for: A single-cell transcriptome atlas of the aging human and macaque retina
Source: Natl Sci Rev. 2020 Aug 25;8(4):nwaa179. doi: 10.1093/nsr/nwaa179 (PMC8288367; doi:10.1093/nsr/nwaa179)

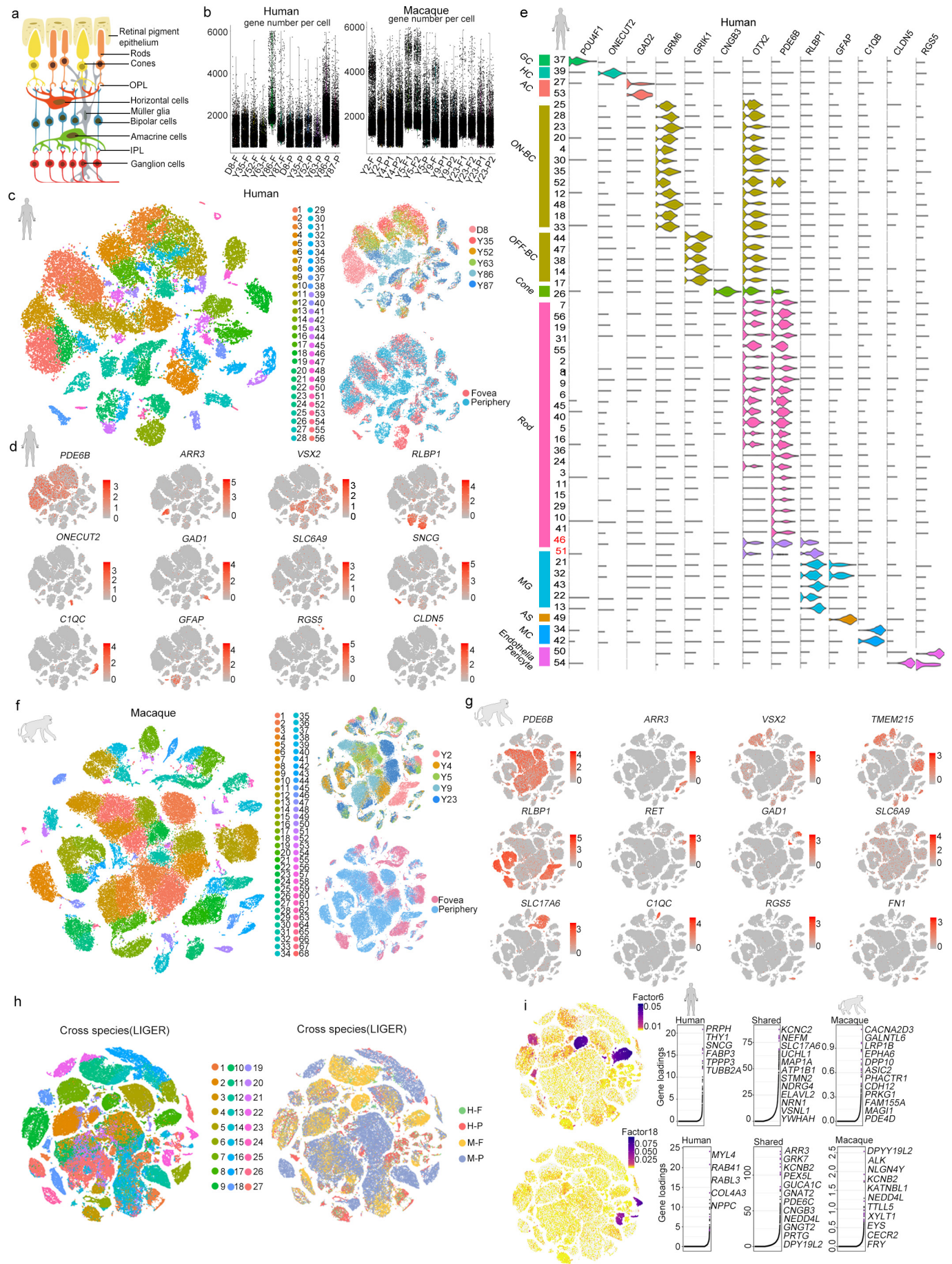

Supplementary Fig1

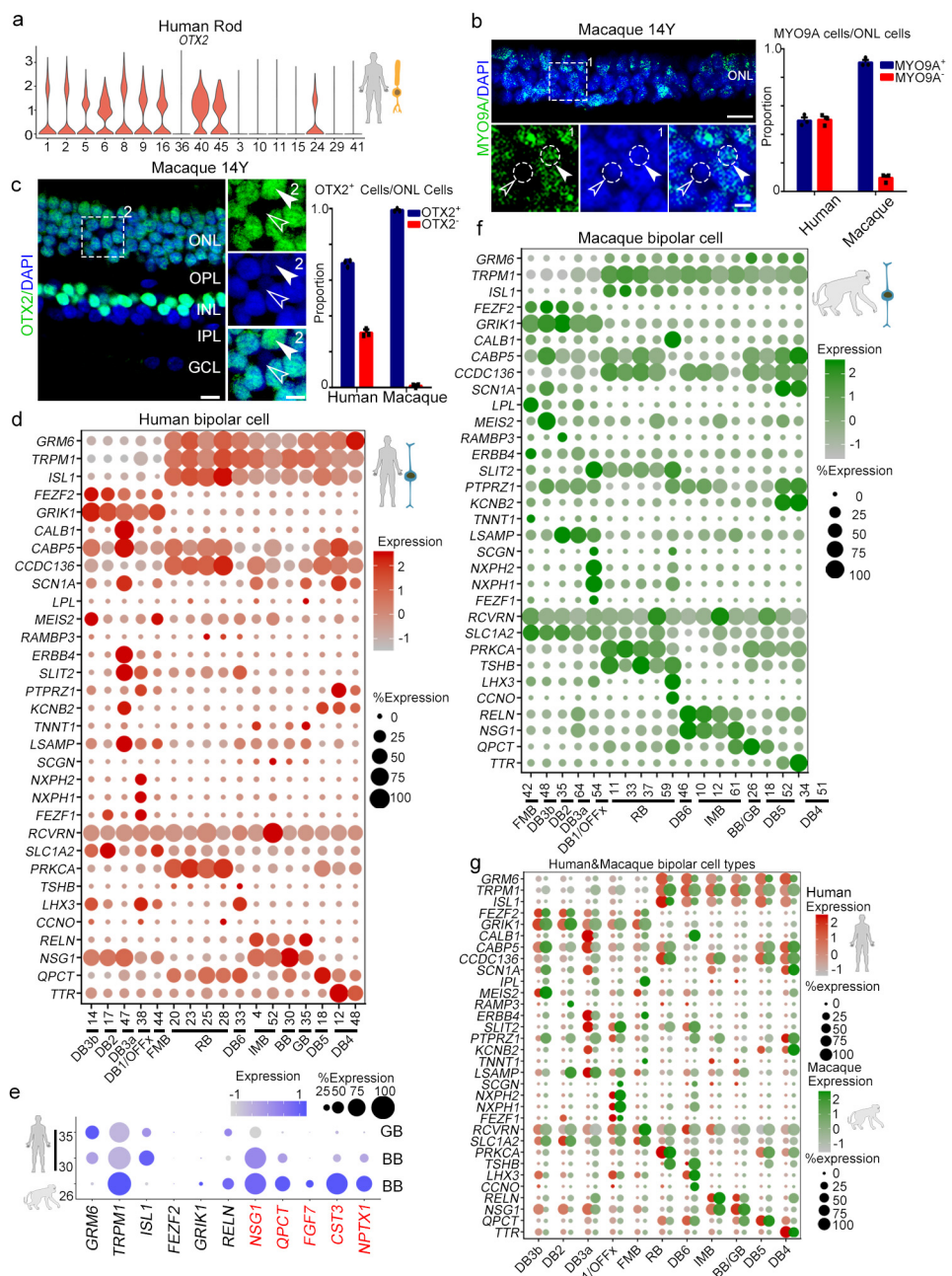

Supplementary Fig2

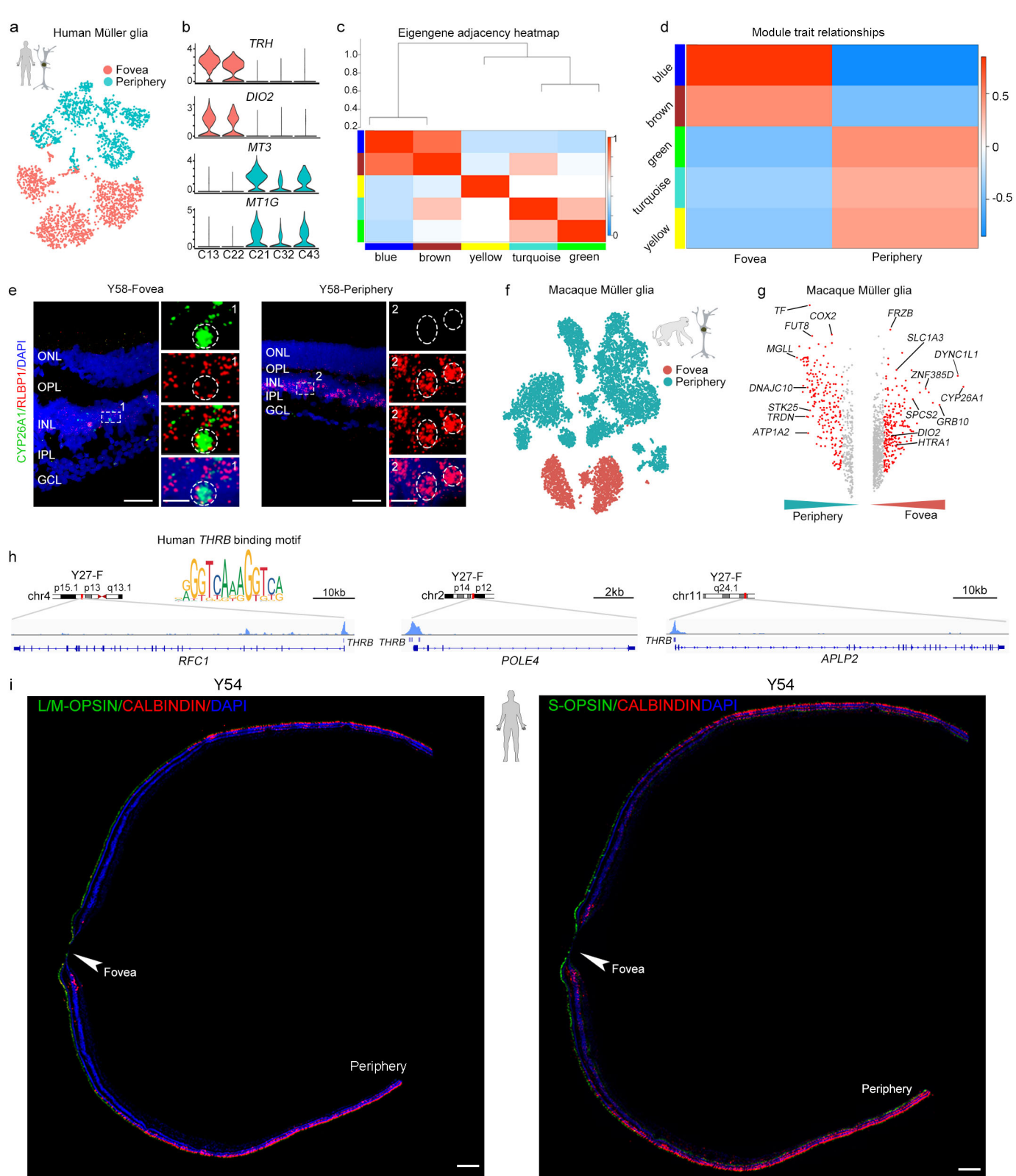

Supplementary Fig3

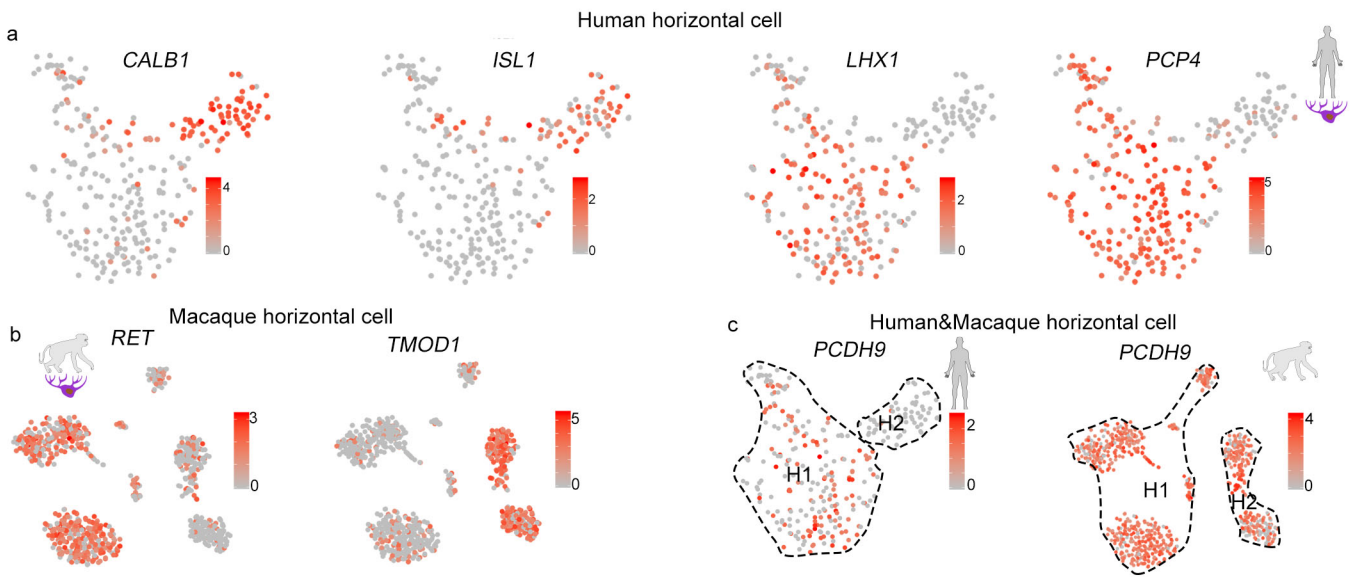



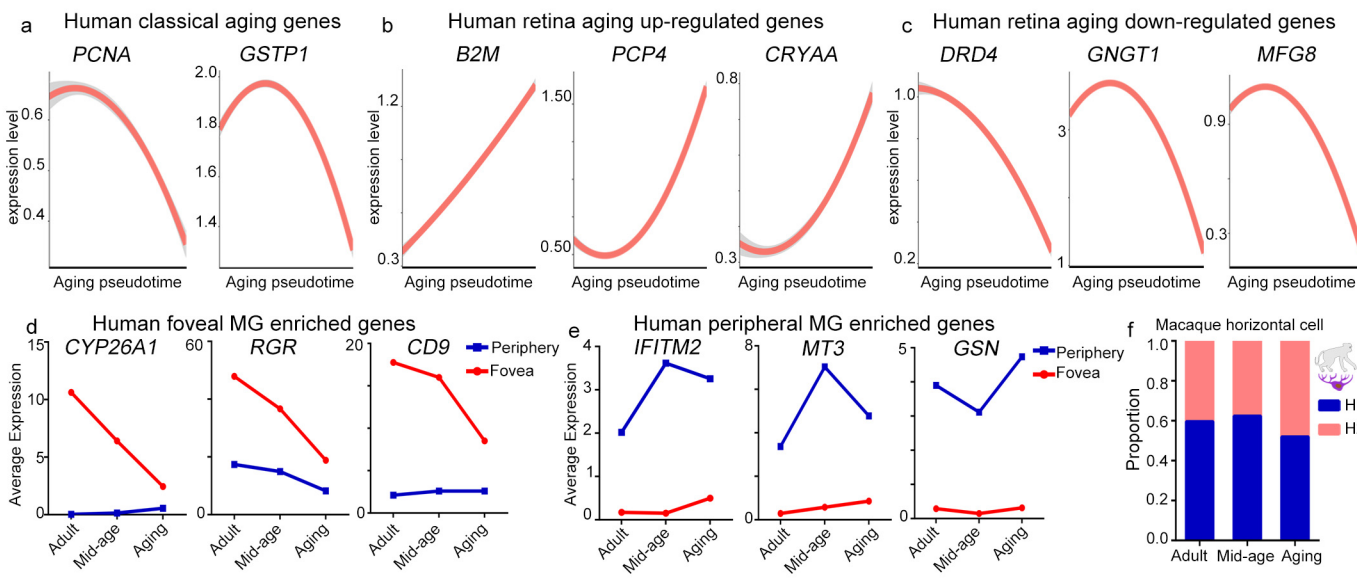

Supplementary Fig6

Supplement: nwaa179_Supplemental_Files [file nwaa179_supplemental_files.zip › nwaa179_Supplemental_Figures.pdf]
